# Assembly of non-unique insertion content using next-generation sequencing

**DOI:** 10.1186/1471-2105-12-S6-S3

**Published:** 2011-07-28

**Authors:** Nathaniel Parrish, Farhad Hormozdiari, Eleazar Eskin

**Affiliations:** 1 Department of Computer Science, University of California Los Angeles, Los Angeles, California, USA

## Abstract

Recent studies in genomics have highlighted the significance of sequence insertions in determining individual variation. Efforts to discover the content of these sequence insertions have been limited to short insertions and long unique insertions. Much of the inserted sequence in the typical human genome, however, is a mixture of repeated and unique sequence. Current methods are designed to assemble only unique sequence insertions, using reads that do not map to the reference. These methods are not able to assemble repeated sequence insertions, as the reads will map to the reference in a different locus.

In this paper, we present a computational method for discovering the content of sequence insertions that are unique, repeated, or a combination of the two. Our method analyzes the read mappings and depth of coverage of paired-end reads to identify reads that originated from inserted sequence. We demonstrate the process of assembling these reads to characterize the insertion content. Our method is based on the idea of segment extension, which progressively extends segments of known content using paired-end reads. We apply our method in simulation to discover the content of inserted sequences in a modified mouse chromosome and show that our method produces reliable results at 40x coverage.

## Introduction

The genetic variation between two individuals may total as much as 8 Mb of sequence content [1]. These variations can vary in size, from single nucleotides up to entire Mb-sized segments of the genome. Variations at the nucleotide level are referred to as single-nucleotide polymorphisms (SNPs), while larger differences spanning an entire segment of the genome are called structural variations (SVs). Structural variations may include instances where a segment of genome is inserted, deleted or inverted in an individual genome. Identifying the variation between two individuals is an essential part of genetic studies. Knowing the content of these variations can help us answer questions such as whether an individual is susceptible to a disease, or why a drug may affect individuals differently. Numerous studies have shown a high correlation between SV and genetic disorders among individuals [2-4]. The variation between one individual (the donor) and another (the reference) is computed by collecting sequence data from the donor, then comparing this sequence to that of the reference. In practice, the reference is typically the NCBI human reference genome (hg17, hg18).

One decade after the emergence of high throughput sequencing (HTS) technology, thousands of genomes have been sequenced using Illumina, ABSOLiD, Solexa, and 454 technology. These technologies are able to sequence a mammalian-size genome in a matter of days, at a cost on the order of a few thousand dollars. This has attracted much attention from both research and industry. HTS has revolutionized the sequencing process, but it has its own drawbacks. Although the technology can generate a very large number of reads in a short amount of time, the length of each read is significantly shorter than is achieved using Sanger sequencing. This limitation has raised a number of challenging computational problems.

Two orthogonal methods have been introduced to detect the variation between an individual diploid donor genome and a haploid reference genome. The first method, known as resequencing, maps all donor reads to the reference [5,6] and uses this mapping information to predict the variation between the donor and the reference [7-11]. In the second method, called assembly, a *de novo* assembler [12-15] is used to assemble the sequence of the donor genome and then detect the differences between the donor and the reference.

One type of structural variation is an insertion of a segment in the donor genome compared to the reference genome. Insertions can be classified as either a unique inserted segment of genome in the donor that does not align to the reference genome, or a copied insertion, where the inserted segment exists in the reference at a different locus.

Kidd et al. 2008 was the first study to tackle the unique insertion problem, and did so by using traditional Sanger sequencing of entire unmapped fosmid clones [16,17]. Unfortunately, this method is costly to apply to HTS data. Many studies in recent years have tried to solve the general SV problem using HTS data [7-11], though these methods were not designed to detect novel insertions. De novo assembly [12-15] can be used to detect the unique and copied insertions, however the high computational cost and memory requirements have made them difficult to use in practice. Moreover, as it is shown by Alkan et.al 2010, *de novo* assemblers have limitations in how accurately they can construct the genome [18]. The only efficient method to assemble unique insertions was introduced by Hajirasouliha et.al [19], which uses paired-end mappings and the unmapped reads to construct the unique insertions using a *de novo* assembler.

In this study we attempt to solve both the unique and copied insertion problems. We will use a hybrid method similar to the method mentioned in [19] using both the reference and a specialized assembler to solve the problem. Our study differs from that carried out by Hajirasouliha et.al [19] in that we are able to successfully assemble insertions comprised of both copied and unique content.

**Figure 1:**
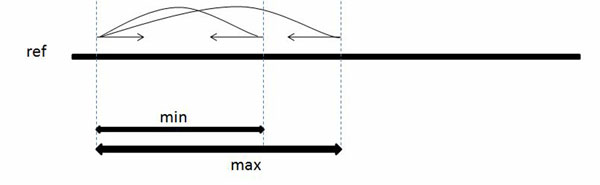
**The sampling of paired-end reads from a genome** The sampling of paired-end reads from a genome in which the distance between sampling positions is at least *min*, but not more than max

**Figure 2:**
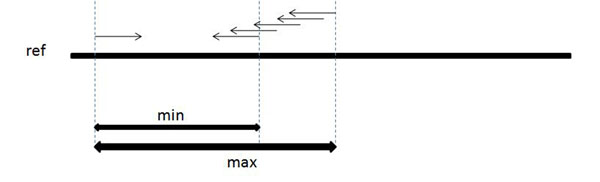
**Estimating the mate position** It is shown that when a mate in a paired-end read is aligned to the reference, one can estimate the position where the second mate aligns

**Figure 3:**
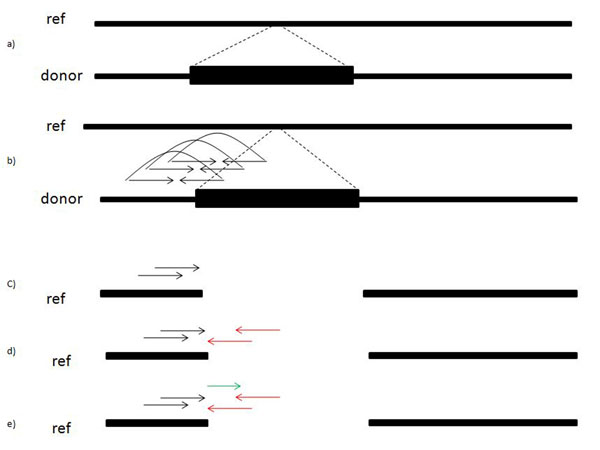
**Illustrating our approach** Each step of our approach is illustrated in this figure. (a) indicates the donor and reference genome, where a segment is inserted in the donor genome. In (b) we show how the reads are sampled from the donor genome. Reads preceding the insertion in (c) map to the reference while their corresponding mate fails to map to the reference. In (d) we show how to use the information from (c) to predict the position of unmapped mates. In the end (e) displays how we can use the (c) and (d) mapping to align additional unmapped reads.

When a paired-end read is sampled from a genome, the distance between the two mates can be modeled as a normal distribution with a mean distance *µ* and standard deviation *σ.* We will further assume for simplicity that the separation distance lies within some well-defined interval, as shown in Figure 1. The first mate is mapped in the forward direction (+) and the corresponding mate is mapped in the reverse direction (–). Given a set of paired-end reads originating from insertion sequences, we will use this fact to ”anchor” one mate in the pair to a known segment of the genome, then find the most likely mapping position of the opposite mate by aligning it with the endpoint of that segment. By finding all such reads that can be anchored and aligned in this way, we are able to discover the content of the insertion sequence beyond the endpoint of the known segment. Iteratively repeating this process allows us to extend these known segments and assemble the insertions. Figures 2 and 3 illustrate this approach.

## Methods

### Notation and definitions

The set of paired-end reads from the donor genome is represented by *R* = {*r*_1_, *r*_2_, *r*_3_, ⋯*r_n_*} where  and  are the forward and reverse strands, respectively, of read *r_i_*.  is the set of positions read  maps to in the reference. *r_i_.loc_k_* is the *k*-th position among all possible mappings of read *i.* ∆*_min_* and ∆*_max_* are the minimum and maximum insert sizes, where ∆*_min_* = *µ* – 3*σ* and ∆*_max_* = *µ* + 3*σ* (three standard deviations from the mean). The set of insertion locations is represented by *Loc* = {*loc*_1_, *loc*_2_, ⋯*loc_m_*}, with *Segs* = {*seg*_0,1_, *seg*_1,2_, ⋯*seg_m_*_–1,_*_m_*} representing the segments of the donor genome between those insertions. The entire donor genome sequence is *Donor.*

We classify reads into the follow five categories:

• *One-end anchored* (*OEA*)*:* Read-pairs in which one mate maps to the reference genome, and one does not.

• *Orphan:* Read-pairs in which neither mate maps to the reference.

• *Concordant:* Read-pairs in which both reads map to the reference, and the distance between their mapped locations is within the range [∆_min_, ∆*_max_*]*.* Furthermore, one mate should map in the forward direction, and one in the reverse.

• *Discordant:* Read-pairs in which both reads map to the reference, but are not concordant.

• *Over-coverage:* Reads which are concordant, but which map to a region with a higher depth-of-coverage than is expected, where the depth-of-coverage in a region is simply the number of reads that map to that region divided by the length of the region.

We will define the following notation for working with slices of strings and arrays: Given an array *A*, *A*[*i* : *j*] denotes a contiguous segment of *A* of length *j* – *i* beginning at position *i.*

We use the ⊕ operation to represent whether or not two strings have an alignment score above some threshold. *s*_1_ ⊕ *s*_2_ = **true** iff Align(*s*_1_, *s*_2_) >*τ*. The exact value of the threshold *τ* varies depending on the context and in all cases is user-configurable, so we leave it as an implicit parameter and omit it from the notation.

The depth-of-coverage for a particular position in the reference is defined as the number of reads that cover that position. We will define *µ_DOC_* as the mean depth-of-coverage across the entire reference genome, and *µ*_*DOC*[*r*]_ as the mean depth-of-coverage across the positions in the reference that are covered by read *r*.

### Assembling the insertion

Given the set of insertion locations *hoc* and the set of reads *R*, our goal is to identify the subset of reads that were sampled from a particular insertion, determine the correct layout of those reads, and finally to decide the consensus value for each position in the insertion. We aim to solve this problem using an iterative approach based on the notion of *segment extension*, which is analogous to building and traversing a path through the string graph [20] simultaneously. We will first present the mathematical foundations of our approach, then describe the optimizations that make this approach practical on common desktop computing hardware.

We begin the insertion assembly process by partitioning the donor genome according to the insert loci *Loc* = {*loc*_1_, *loc*_2_, ⋯*loc_m_*}*.* This results in a set of segments *Segs* = {*seg*_0,1_, *seg*_1,2_, ... *seg*_*m* – 1,_*_m_*}, where *seg_i_*_,_*_i_*_+1_ represents the segment of the donor genome between insertion loci *i* and *i* + 1. For each segment *seg_i_*_,_*_i_*_+1_, we attempt to assemble insertions *i* and *i* +1 by *extending* the segment at each endpoint using an iterative process. For the sake of simplicity, we will formulate only extension in the forward direction.

The segment extension method is based on identifying a set of reads which have a high likelihood of covering a particular position *pos* in the donor genome, where *pos* lies at the edge of some segment *seg.* Identification of this set occurs in two passes. The first pass is performed only once, and selects reads from *R* which are likely to have been sampled from any insertion in the donor genome. We refer to the result of this first pass as the *insertion read set.* The second pass is performed for every position *pos*, and further filters the insertion read set to select reads that are likely to cover position *pos.* We refer to the result of the second pass as the *covering set* for position *pos.* Once we identified the covering set, we decide the value of *Donor*[*pos*] by finding the consensus among all reads in the set. We then move to position *pos* + 1 and repeat the process.

#### Insertion read set

Consider a paired-end read *r* in which one mate covers the insertion and the other mate does not. In the case that the insertion sequence is unique, it follows that *r*^–^*.loc* = ∅ or *r*^+^.*loc* = ∅ (∅ being the empty set), categorizing the read as OEA. In the case where the insertion sequence is copied, then both mates will map somewhere in the reference, however the distance between them is unlikely to be consistent with the expected insert size (|*r*^+^*.loc* – *r*^–^*.loc*| < ∆*_min_* or |*r*^+^*.loc* – *r*^–^*.loc*| > ∆*_max_*)*.* In this case the read will be categorized as Discordant.

Now consider a paired-end read *r* in which both reads cover the insertion. If the insertion sequence is unique, then neither mate mate will map to the reference and the read will be categorized as an Orphan read. On the other hand, if the insertion sequence is copied, then both mates will map to some region in the reference, and the distance between them will be consistent with the expected insert size. However, if we calculate the depth of coverage in this region, we will find it to be higher than the sequencing coverage. These reads will be categorized as over-coverage.

Based on this analysis, we define the following four functions:(1)(2)(3)(4)

We now construct a subset *IRS* = {*r* ∈ *R* : IsOEA(*r*)| IsOrphan(*r*)| IsDiscordant(*r*)| IsOverCoverage(*r*)}, representing the insertion read set. Note that the IsOverCoverage function is designed such that we select the appropriate fraction of reads from an over-coverage region. For example, if a region has mean read-depth 2*µ_DOC_*, we are only interested in 50% of the reads from that region. This is captured by the probabilistic function.

#### Covering set

Consider a position in the donor genome *Donor*[*pos*] belonging to an insertion and for which the correct nucleotide assignment is unknown. Our goal is to determine the exact set of reads *CS_pos_* that cover *Donor*[*pos*], which we will refer to as the *covering set.* Assume that *Donor*[*j*] is known for all *j* ∈ [(*pos* – 2*l* – ∆*_max_*),*pos*] and consider a paired-end read *r* = (*r*^+^, *r*^–^)*.* We assert that if *r*^–^ covers *Donor*[*pos*], in other words *r* ∈ *CS_pos_*, then the following conditions must hold:

1. *r*^+^ covers some set of positions in *Donor*[*pos* – *I* – ∆*_max_* : *pos* – ∆*_min_*].

2. If *r*^–^[*ext*] covers *Donor*[*pos*], then *Donor*[*pos* – *ext* – 1 : *pos* – 1] ⊕ *r*^–^[0: *ext* – 1].

The region of the donor genome denoted by *Donor*[*pos* – *I* – ∆*_max_* : *pos* – ∆*_min_*] is referred to as the *anchor window*, and so reads that meet condition 1 are considered *anchored.* Reads that meet condition 2 are referred to as *extending* reads. We will capture these conditions formally in two functions Φ*_A_* and Φ*_E_* (*anchors* and *extends*, respectively), defined as follows:(5)(6)

**Figure 4:**
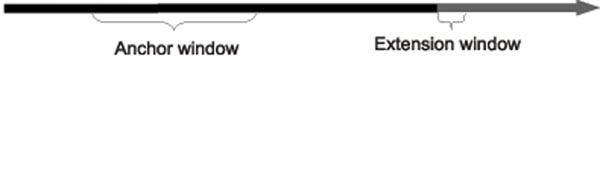
Defining the anchor and extension windows

**Figure 5:**
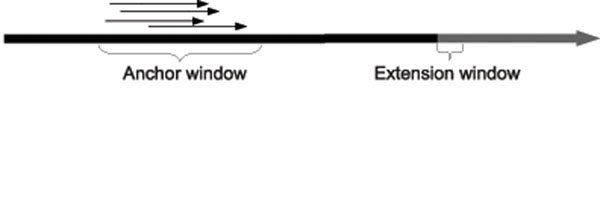
Identifying anchoring reads

**Figure 6:**
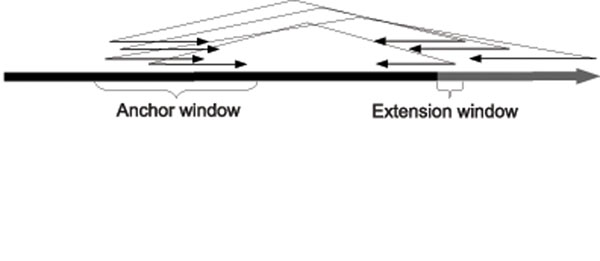
Identifying extending reads

Refer to Figures 4, 5, and 6 for an illustration of the process identifying the covering set. Note that for our purposes, small values of *ext* are not informative, as there is a relatively high probability, given two short strings *s*_1_ and *s*_2_, that *s*_1_ ⊕ *s*_2_. Therefore we will further require that *ext* >*κ*, where *κ* is user-configurable. In practice, given a paired-end read it is not known a priori which mate is the forward strand and which is the reverse. During construction of the covering set we therefore test both orientations and settle on one should it be found to meet the two conditions.

We can now compute an approximation of *CS_pos_* as follows: . We note that this is only an approximation, as the repetitive nature of genomic sequence dictates that there will be reads in  that do not truly cover *Donor*[*pos*]*.* Further-more, our choice of *κ* as a lower threshold means there will be reads in *CS_pos_* that are not in *.*

**Figure 7:**
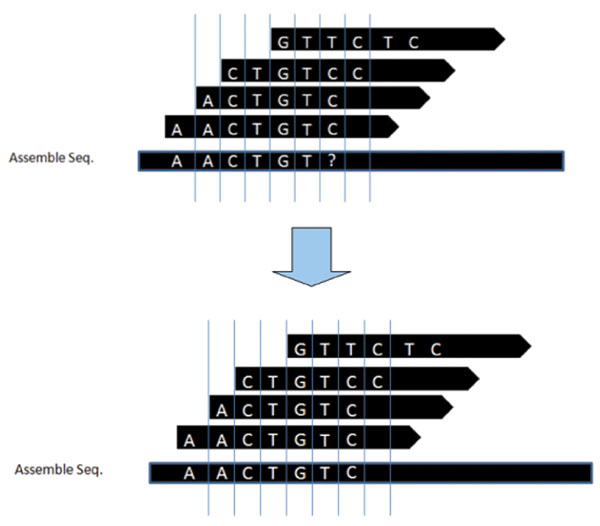
**Extending the segment** Indicating how we can compute the 7th element in the donor which is indicated by ? in the left figure. Using the mapping reads we can replace the ? with C

Using the covering set we can now decide the value of *Donor*[*pos*] as follows, where *ext_s_* is the value of *ext* computed for read *s.* Note that this is merely a formal statement of the standard consensus problem. Refer to Figure 7 for an illustration of this.(7)

Once the value of *Donor*[*pos*] is known, we can iteratively repeat this process for *pos* + 1, *pos* + 2,.... Recall that we initially assumed that the value of *Donor* [*j*] is known for all *j* : *i* – 2*l* – ∆*_max_* <*j* <*i.* This will always be the case at the boundaries of the insertion, providing a base from which we can iterate. The iteration terminates when there is insufficient consensus to decide the value of *Donor*[*pos*]*.* That is,  , where *ε* is a user-defined threshold.

Algorithm 1 illustrates the high-level algorithm. Once this iterative process has been applied to each segment, we are left with a set of extended segments . In order to assemble the complete donor genome, we compute the overlap of each adjacent pair of segments. Pairs with a low overlap score indicate that only a portion of the insertion between them was assembled, in which case it may be possible to revisit the insertion using a more relaxed set of alignment functions. Pairs with high overlap scores indicate a successful insertion assembly and may be merged into a single contig.

Note that in algorithm described above, at each iteration we selected the value that is the consensus of all reads in the covering set. In general, this approach works well when applied to non-repetitive insertion sequences. For insertion sequences that are repetitive, however, there will be multiple values of c for which , where *ε* is suitably large to eliminate the effect of read errors. In this case we say that our iterative algorithm has encountered a *divergence*, and we proceed to branch and explore each supported value of c. Ultimately, each branch will return a set of hypothetical sequences. In attempting to select the most probable sequence from this set, we reason that an ideal assembly of all insertions would account for every read in *IRS.* Therefore when assembling each individual insertion, we select the hypothesis that accounts for the greatest number of reads.

We assume that the locations of the insertions are provided to us as input to the assembly method. There are two main methods for determining these locations that have been presented in previous work:

1. *Using existing SVs frameworks:* Many efficient tools have been developed in past few years to detect the SVs efficiently and accurately [8,10]. We can use the output of their methods as the input to our algorithm.

2. *Clustering the OEA Reads:* OEA reads are indicator of unique insertion, we will cluster the OEA reads and pick the cluster set which has the most number of OEA reads. Clustering the OEA reads will increase our confidence level if an insertion has occurred in the donor genome. Furthermore, it will reduce the estimated number of unique insertions in the donor genome, which follows the maximum parsimony [19].

### Optimization

Given a donor genome containing a total length *L_I_* of all insert content, a naïve implementation has a running time that is dominated by the insertions assembly step, with a running time of . For every position *pos* in each insertion, we must search through |*IRS*|, computing Φ*_A_* and Φ*_E_* for each read, to identify the approximate covering set . Note that as |*IRS*| will be dictated by *L_I_*, and  will be fairly constant, this can be roughly simplified to .

In order to reduce the computational complexity of this search problem, we make use of recent methods developed for read-mapping applications using the Burrows-Wheeler Transform (BWT) [21]. While we will not discuss the implementation details, the advantages of using a BWT can be summarized as follows. Given two strings *x* and *y*, we would like to find all instances of string *y* in *x*, allowing for *d* mismatches. While a naïve search algorithm would require *O*(|*x*||*y*|) operations, using a BWT we can achieve this in only *O*(ℓ*^d^*|*y*|) operations, irrespective of |*x*|, where ℓ is the size of the language (4 in our case). Furthermore, unlike the common suffix tree-based approaches, the BWT can be represented compactly, making this approach feasible on standard desktop hardware.

In read-mapping applications such as *BWA* and *Bowtie*[22,23], *x* is the reference genome, and *y* is an individual read. We instead set , the concatenation of all forward-end reads, and search for substrings *s* of the anchor window. Given a function BWTSearch that returns the set of matching indices, we can now use the BWT to locate all anchored reads:

That is, if read *r_j_* is anchored, then one of the calls to BWTSearch should return the index *jl.* The key difference here is that computing the set on the left requires computing Φ*_A_* for all reads in *R'*, while the set on the right can be computed using only ∆*_max_* – ∆*_min_* (the number of substrings of length *l* in the anchor window) calls to BWTSearch.

We also note here that as each insertion is assembled independently, it is straightforward to parallelize our approach on multiple processors. Once the insertion read set *IRS* has been generated, it can be read by all processes on a single machine or cloned on each machine in a cluster. Each process is then assigned a single segment to extend. Furthermore, the construction of *IRS* itself can be parallelized simply by dividing up the set of reads among multiple processes.

## Results

In this part of the paper we will report the accuracy of our method in assembling the insertions. We designed a simulated framework in which the reference genome is the C57BL/6J (NCBI m37) chromosome 17 and the donor genome is simulated by inserting sequence segments into the reference genome. Unique insertions were generated using a uniform distribution over the four bases. Copied insertions were generated by choosing a uniformly random position in the genome and duplicating the content at that position. The mean size of the inserted segments is 2kbps, with a standard deviation of 200bp. We generate a set of reads from the donor genome using MetaSim [24], using a read length of 36 and a mean insert size of 200bp with a standard deviation of 25bp. We generate reads at 40X coverage. Moreover, we vary the number of inserted segments from 10-1000. We calculate the accuracy of our method by counting the number of insertions that were assembled correctly within some small margin of error (an edit distance of 10bp was used in the results shown). Table 1, shows the results of this calculation, confirming that our method maintains high reliability as the number of insertions grows. In these results, each insertion contains equal parts unique and non-unique content, generated by copying a segment of the reference genome and inserting a unique segment. The decrease in accuracy as the number of insertions grows can be attributed to the increase in the number of reads contained in the Insertion Read Set. As the size of this set grows, the probability of selecting the wrong reads during segment extension also increases. Our results demonstrate, however, that this effect is fairly small.

**Table 1:** Accuracy of our method at varying numbers of insertions, from 10 to 1000

#Insertion	Accuracy(%)	Standard Deviation(%)
10	98.00%	4.47%
50	92.80%	1.79%
100	94.20%	2.49%
500	91.64%	1.04%
1000	89.92%	0.77%

Accuracy of our method at varying numbers of insertions, from 10 to 1000. Each insertion contains both unique and non-unique content.

**Figure 8:**
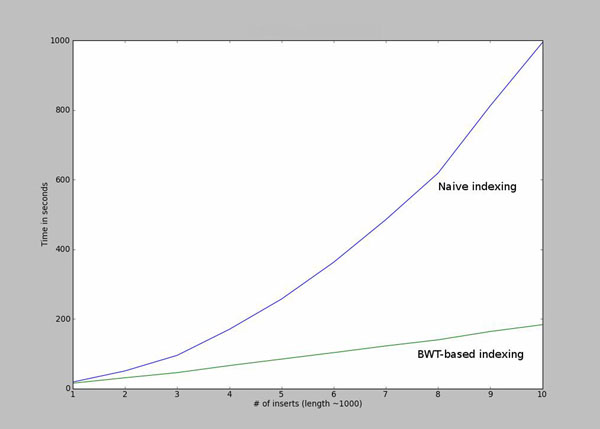
Comparison between the naive search method and the BWT search method

In Figure 8 we show that the running time of the algorithm increases quadratically if we apply the naive indexing algorithm. Using the Burrows-Wheeler Transformation discussed in the Optimization section results in a running time that grows linearly.

**Figure 9:**
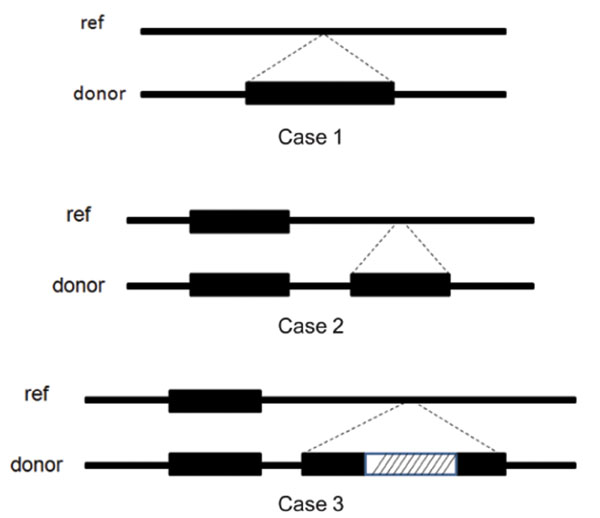
Three different categories of insertions

In order to test our method in different insertion categories, we run our method on three different cases as shown in Figure 9. Case 1 is where the insertion is unique and the sequence is inserted in a unique region in the reference. In Case 2 the insertion is copied but it is inserted in a unique region. Case 3 is similar to case 2, where the insertion is copied but contains a unique segment as well. Table 2 indicates the assembly accuracy for 1000 insertions in the 3 different categories. In the first case we are testing how accurately our method can assemble the unique insertions. This is the simplest case among the three. In the second case, not only we are testing our assembly accuracy, but our success is also an indication of how well we can detect the set of over-coverage reads. High accuracy in the second case is not only important for insertion assembly, but it can also be widely used in the CNV detections. In case 3, in addition to the complexity in case 2 and case 1, we have to deal with the case where there is an insertion inside another insertion. As the results indicate our method maintains high accuracy as the complexity of the insertions grows, which suggest we can apply our method to any insertion assembly problem, without any assumptions as to the type of the insertions.

**Table 2:** Accuracy of our method in 3 different categories

Category	Accuracy(%)	Standard Deviation(%)
Case 1	98.40%	0.23%
Case 2	92.06%	0.98%
Case 3	89.92%	0.77%

Accuracy of our method in 3 different categories. In Case 1 both the inserted sequence and the region in the reference is unique. In Case 2 the copied sequence is inserted in a unique region in the reference, while in Case 3 the copied insertion contains a unique insertion as well. For each case, 5 simulations were performed.

## Discussion

Detecting structural variation (SVs) between two individuals has been studied widely in the past few years. Although detecting the presence of SVs is an important problem, assembling the actual sequence of the SV accurately is invaluable. While high throughput sequencing (HTS) has revolutionized genomics by giving us the opportunity to cost effectively sequence many individuals, this technology limits the extent to which reference-based assemblers can discover the content of inserted sequences. In this study we addressed the insertion assembly problem using paired-end HTS data. While previous methods were focused on assembling the content of unique insertion sequences, and thus are not able to assemble insertions containing copied regions, our method is able to assemble both copied and unique insertions. Furthermore, it is independent of any de novo assemblers, and as such it can be used as a stand alone tool to assemble insertion sequences. We have shown that at 40X coverage we can assemble the insertions with very high accuracy. Finally, we have demonstrated the practicality of our approach by presenting both algorithmic optimizations and parallelization opportunities that make this method feasible even for mammalian-size genomes.

## Competing interests

The authors declare that they have no competing interests.
